# Design and Psychometric Evaluation of a Nursing Care Rationing Instrument

**DOI:** 10.1002/nop2.70933

**Published:** 2026-10-16

**Authors:** Mohammad Mehdi Mohammadi, Mohammad Mohammadi, Sara Bakhtiyari

**Affiliations:** ^1^ Department of Medical‐Surgical Nursing, School of Nursing & Midwifery Kermanshah University of Medical Sciences Kermanshah Iran; ^2^ Student Research Committee Kermanshah University of Medical Sciences Kermanshah Iran

**Keywords:** instrument design, measurement properties, nursing care rationing, psychometric evaluation, scale development

## Abstract

**Aim:**

The present study aimed to design and validate an instrument for measuring nursing care rationing based on the limitations of existing instruments.

**Design:**

This study employed an instrument design and psychometric evaluation methodology.

**Methods:**

In the initial item generation phase, an exploratory qualitative study using conventional content analysis was conducted through individual semi‐structured interviews with 15 clinical nurses to extract rationing dimensions and formulate an initial item pool alongside literature reviews. Following face and content validity assessments, the psychometric properties of the instrument were evaluated among 220 clinical nurses recruited from educational hospitals. Construct validity and dimensionality were examined using exploratory factor analysis (EFA), Exploratory Graph Analysis (EGA) and Mokken Scale Analysis (MSA). Stability was further assessed via a Monte Carlo simulation (1000 replications), and reliability was evaluated using internal consistency indices and test–retest reliability (*n* = 30).

**Results:**

The final instrument comprises 12 items accounting for 71.97% of the total variance in exploratory factor analysis. A single‐factor dimensionality was supported by EGA (with 100% item stability across 500 bootstrap iterations) and Mokken Scale Analysis (overall scalability coefficient H=0.762). Monte Carlo simulation (1000 replications) demonstrated high structural replicability (mean Tucker's congruence coefficient =1.00, statistical power =100%). Reliability assessments demonstrated high internal consistency (Cronbach's α=0.953, McDonald's ω=0.968) and test–retest stability (ICC=0.969, 95% CI: 0.953–0.985).

**No Patient or Public Contribution:**

Patients or the public were not involved in the design, conduct, reporting, or dissemination plans of this research.

## 1 Background

Nurses, as the largest healthcare workforce in hospitals, play a fundamental role in delivering essential care to patients (Zhao et al. 2020; Shamsi and Peyravi 2020). The nursing profession is recognized as a demanding and crucial occupation (Harms 2021). At its core, nursing is defined by care, which serves as a primary distinguishing factor from other health‐related professions (Maghsoud et al. 2022).

Within the challenging landscape of the nursing profession, a significant and persistent stressor for nurses is a heavy workload. This issue is particularly pronounced in departments facing resource limitations, including nurse shortages. Consequently, each nurse is responsible for the care of a number of patients exceeding standard ratios. This increase in the patient‐to‐nurse ratio leads to a substantial rise in workload pressure on nurses, consequently diminishing their time and capacity to provide thorough and sensitive care to each patient (Asjanti and Irbantoro 2022; Papastavrou et al. 2014). As a result, nurses are inevitably compelled to ration the nursing care they provide (Uchmanowicz et al. 2020).

Nursing care rationing refers to the allocation of nursing care resources in a manner that results in some patients receiving less care than required. This phenomenon typically occurs due to resource scarcity, including shortages of nurses, equipment, or time. Consequently, nurses are compelled to make difficult decisions regarding which care to provide to which patients (Hegney et al. 2019).

Conceptually, nursing care rationing must be clearly distinguished from related constructs. Unlike “workload,” which describes the volume of demands placed on a nurse, rationing is the active, cognitive decision‐making and prioritization process resulting from that excessive workload. Furthermore, while terms like “missed care” or “unfinished care” describe the end‐result – the actual omission of required care – implicit rationing focuses on the behavioural process and the challenging ethical choices that lead to those omissions (Scott et al. 2019). Nursing care rationing is not an uncommon issue (Renner et al. 2022) and is considered a global problem within healthcare systems, posing a significant challenge to ensuring patient safety and promoting quality of care (Uchmanowicz et al. 2020). Indeed, the rationing of care arises when nurses lack sufficient resources to ensure adequate patient care; therefore, nurses are obligated to prioritize their tasks (Kalánková et al. 2020). Definitions provided regarding the concept of nursing care rationing also indicate an attempt to convey that when a lack of resources renders the provision of all necessary care impossible, nursing activities are either partially or completely omitted, whether intentionally or unintentionally (Zhao et al. 2020).

There are numerous reasons for rationing care (Uchmanowicz et al. 2020). Reports indicate that rationing occurs when the available resources are insufficient to provide care to patients (Tomaszewska et al. 2021). The nurse's work environment is considered an organizational characteristic that either promotes or undermines the nurse's professional autonomy (Zhao et al. 2020). Accordingly, the literature points to factors such as staff shortages, burnout and stress as antecedents of nursing care rationing. The issue of nursing staff shortages affects many healthcare institutions globally and is considered a primary antecedent of nursing care rationing (Shamsi and Peyravi 2020; Uchmanowicz et al. 2020). Workplace characteristics, unfavourable working conditions, teamwork and safety climate, as well as staff characteristics, documentation, high patient loads, insufficient time and excessive responsibilities can all contribute to the rationing of nursing care (Tomaszewska et al. 2021; Ausserhofer et al. 2021). All of these interrelated human and organizational factors contribute to the socio‐ecological phenomenon of implicit nursing care rationing (Renner et al. 2022).

Adverse effects of nursing care rationing include increased mortality rates, falls from bed during hospitalization, diminished quality of care, the development of pressure ulcers and a higher incidence of nosocomial infections (Uchmanowicz et al. 2020). Reports indicate that care rationing is a predictor of reduced care quality, decreased patient satisfaction, an increased number of adverse events and lower job satisfaction among nurses (Uchmanowicz et al. 2020; Witczak et al. 2021). The prioritization of care activities and the omission of specific tasks place patients at risk of having their educational, emotional and psychological needs unmet. Nurses who experience a high degree of nursing care rationing in their work are susceptible to emotional exhaustion stemming from burnout (Uchmanowicz et al. 2020).

Another consequence of nursing care rationing is the issue of missed care or incomplete care. Evidence suggests that the phenomenon of missed care contributes to feelings of guilt, a sense of powerlessness in providing necessary patient care and fear of being victimized among nurses (Scott et al. 2019).

Unfortunately, precise statistics regarding the rationing of nursing care in Iran and globally are currently unavailable (Shamsi and Peyravi 2020). The quantification of data on any given subject necessitates the existence of a suitable instrument. The lack of accurate statistics on nursing care rationing stems from the absence of an appropriate tool for its measurement to date. Until a suitable instrument is designed and developed, obtaining precise statistics on the rationing of nursing care will remain unattainable.

A review of the existing literature reveals several instruments for measuring nursing care rationing, such as the perceived implicit rationing of nursing care questionnaire developed by Yuwanto et al. with the aim of measuring implicit nursing rationing. The target population for this instrument comprised nurses working in two public hospitals in the Jember Regency of Indonesia (Yuwanto et al. 2023). This questionnaire is a translation of the PRINCA instrument, which itself is derived from the Basel Rationing of Nursing Care Scale (BRENCA) developed by Schubert in Switzerland. This instrument primarily focuses on nurses in Indonesia and is not a suitable measure for nursing care rationing in Iran because Iran and Indonesia differ in terms of their conceptual, cultural, social and economic contexts.

Another instrument identified was the Basel Extent of Rationing of Nursing Care instrument validation questionnaire, designed by Schubert in 2007. This instrument was studied in Switzerland with the aim of measuring implicit rationing of nursing care. It includes 20 questions about essential nursing tasks across five dimensions: (a) activities of daily living, (b) care and support, (c) rehabilitation and education/training, (d) monitoring and safety and (e) documentation (Schubert et al. 2007). Since this instrument was evaluated in a country with a different cultural, social, economic and educational context, it is not applicable in Iran.

The subsequent instrument was the Individual Workload Perception Scale questionnaire. This tool was developed and administered between June 1st and September 30th, 2009, by Shu‐Yuan Lin and colleagues using convenience sampling of 344 nurses providing direct patient care at a regional teaching hospital in southern Taiwan. This questionnaire is designed to measure nurses' perceptions of their workload and work environment (Lin et al. 2011). However, besides being implemented in a country with a different cultural context and resources compared to Iran, its foundation lies in measuring the workload of nursing personnel and does not assess our topic of interest, which is the rationing of nursing care.

Considering the limitations of existing scales, a critical research gap remains in the literature. Current tools either measure related but distinct concepts – such as nursing workload rather than the active cognitive process of care rationing‐ or they were developed in healthcare systems with vastly different cultural, socio‐economic and organizational dynamics, rendering them inapplicable to the Iranian context. To address this substantial gap, the present study aimed to design and psychometrically evaluate a culturally tailored, context‐specific Nursing Care Rationing Instrument for the Iranian nursing workforce. Developing this valid and reliable tool is a fundamental prerequisite for accurately quantifying care rationing, thereby providing a foundation for future research and evidence‐based nursing management in Iran.

## 2 Methods

### 2.1 Study Design and Item Generation

The present research was an instrument design study conducted with the aim of developing and validating a nursing care rationing instrument for clinical settings. An inductive and deductive methodology was employed for the generation of items (Boateng et al. 2018). The inductive phase consisted of individual qualitative interviews exploring the lived experiences of nurses regarding care prioritization, time scarcity and resource allocation. The interview data were transcribed and analysed using conventional content analysis to establish a clear conceptual foundation. Based on these qualitative findings, nursing care rationing was conceptualized as a complex process of making clinical decisions necessitated by severe systemic constraints and high patient volumes. It involves the cognitive prioritization and equitable allocation of limited care resources among multiple patients. Nurses are inevitably compelled to make critical clinical compromises. They may deliberately omit less urgent supportive services, sacrifice educational duties or delegate fundamental care tasks to companions to preserve essential medical interventions. This conceptualization reflects a continuous struggle where the disparity between excessive workload and available capacity prevents nurses from fully satisfying the comprehensive care demands of their patients. Simultaneously, the deductive phase involved a comprehensive review of relevant literature to capture existing theoretical dimensions. An initial item pool was subsequently formulated based on this extracted conceptualization and the literature review. Finally, overlapping or redundant items were merged and removed to finalize the preliminary draft of the instrument.

### 2.2 Participants and Sampling Framework

To ensure methodological rigour and avoid sample contamination across developmental stages, distinct and mutually independent cohorts of participants were recruited for each phase of the study, as outlined below:

- *Phase 1*: *Item Generation* (*Qualitative Phase*): Fifteen clinical nurses with diverse clinical backgrounds were selected using purposive sampling with maximum variation (considering ward type, shift patterns and clinical experience) until data saturation was reached.
- *Phase 2*: *Face Validity Assessment*: An independent group of 10 registered clinical nurses actively engaged in direct patient care was recruited via convenience sampling to assess qualitative and quantitative face validity (item impact scoring).
- *Phase 3*: *Content Validity Assessment*: A panel of 10 university faculty members and academic specialists in nursing management and instrument development was purposively selected to evaluate qualitative content clarity, Content Validity Ratio (CVR) and modified kappa‐based Content Validity Index (CVI).
- *Phase 4*: *Initial Reliability Assessment* (*Pilot Testing*): Prior to factor extraction, an independent convenience sample of 30 clinical nurses was recruited to examine preliminary item‐total correlations and initial internal consistency.
- *Phase 5*: *Construct Validity and Scale Dimensionality*: A separate, independent cohort of 220 clinical nurses was recruited using convenience sampling from multiple inpatient departments to undergo Exploratory Factor Analysis (EFA), Exploratory Graph Analysis (EGA) and Mokken Scale Analysis (MSA). Data from this cohort were also utilized to compute McDonald's omega for internal consistency. None of these participants had been involved in the preceding qualitative, face validity or pilot testing phases.
- *Phase 6*: *Final Reliability Assessment*: An independent subgroup of 30 clinical nurses was recruited to evaluate the final internal consistency (Cronbach's alpha and Average Inter‐item Correlation) and temporal stability (test–retest reliability completed twice with a two‐week interval).

### 2.3 Study Setting and Data Collection Procedures

This study was conducted across five major tertiary educational and teaching hospitals affiliated with Kermanshah University of Medical Sciences. These hospitals serve as provincial referral medical centers. Data collection was carried out systematically across sequential phases:

- *Phase 1*: *Qualitative Item Generation*: Individual, face‐to‐face, semi‐structured qualitative interviews were conducted by the first author with 15 clinical nurses. To ensure psychological safety, confidentiality, and minimal disruption to clinical workflow, all interviews took place during off‐peak hours or at shift changeovers in a quiet, private room within the respective clinical ward (such as a private head nurse consultation office or an unoccupied staff break room). Each interview lasted 35 to 65 min. With the participants' explicit consent, sessions were audio‐recorded, anonymized and transcribed verbatim within 24 h of completion.
- *Phases 2* & *3*: *Face and Content Validity*: Standardized evaluation forms containing the operational definition of nursing care rationing alongside the item pool were distributed in person and electronically. Completed appraisal forms were retrieved within a designated two‐week timeframe. All responses were treated confidentially and entered anonymously into an aggregated scoring matrix.
- *Phase 4*: *Initial Reliability Assessment*: The provisional instrument (retaining items approved post‐content validity) was administered in person to 30 clinical nurses. Responses were collected in sealed envelopes and analysed to evaluate preliminary item discrimination and initial Cronbach's alpha, guiding the refinement of the item pool prior to the large‐scale survey.
- *Phase 5*: *Construct Validation Survey*: Self‐administered paper questionnaires were distributed in person by the researchers across rotating clinical shifts (morning, afternoon and night shifts) to ensure representative sampling from diverse clinical environments. To uphold confidentiality and mitigate social desirability bias regarding care omissions, questionnaires were distributed in sealed, unidentifiable envelopes. Participants completed the survey during off‐duty rest periods and deposited the sealed questionnaires into designated drop‐boxes stationed in nursing administrative units. A total of 220 registered nurses from across diverse hospital departments participated in this phase, representing general internal medicine and medical subspecialties (*n* = 68, 30.9%), surgical departments (*n* = 53, 24.1%), emergency units (*n* = 36, 16.4%), paediatric and obstetric units (*n* = 28, 12.7%), adult and paediatric intensive care units (ICU, CCU, PICU, NICU; *n* = 22, 10.0%) and specialized outpatient units (*n* = 13, 5.9%).
- *Phase 6*: *Final Reliability Assessment*: To evaluate final internal consistency and temporal stability without compromising anonymity, a two‐stage alphanumeric coding procedure was employed for a separate cohort of 30 nurses. Participants generated a self‐selected, non‐identifying personal identifier on the initial questionnaire. Two weeks later, the scale was re‐administered to the same cohort using the matching identifier, allowing exact paired tracking while keeping participant identities fully anonymous.

### 2.4 Face Validity

*Quantitative Assessment of Face Validity*: In this stage, the Item Impact method was employed according to the steps outlined in Table 1 to determine the quantitative face validity.

**TABLE 1:** Stages of the item impact method in assessing face validity.

Stage	Stage description
1	Each item in the target questionnaire is rated using a five‐point Likert scale (Very Appropriate = 5, Appropriate = 4, Moderately Appropriate = 3, Inappropriate = 2, Very Inappropriate = 1).
2	Ten nurses independently evaluated each questionnaire item based on this Likert scale. The “Item Impact Score” for each item was then calculated separately using the following formula: Item Impact Score = Frequency (%) × Suitability (Importance) In this formula, Frequency refers to the percentage of respondents who assigned a score of 4 or 5 to the item, while Suitability represents the mean score given to the item.

*Note:* An impact score of 1.5 or higher indicates that the item is appropriate, whereas a score below 1.5 suggests that the item requires revision or modification.

*Qualitative Assessment of Face Validity*: Qualitative face validity was evaluated by assessing the items for difficulty level (comprehension of words and phrases), irrelevance (mismatch with the intended construct), and ambiguity (potential misinterpretations) and refining them based on participant feedback.

### 2.5 Content Validity Assessment

To assess content validity, both qualitative and quantitative methods were employed (Polit and Yang 2016; Colton and Covert 2007).

#### 2.5.1 Qualitative Content Validity Assessment

The expert panel evaluated the qualitative content validity with a focus on grammar, appropriate wording, correct item placement, scaling format, simplicity and conceptual clarity.

#### 2.5.2 Quantitative Content Validity Assessment

The quantitative assessment involved two sequential steps. Initially, the Content Validity Ratio (CVR) was calculated, following which certain items were eliminated based on the results. Subsequently, the Content Validity Index (CVI) was determined using the adjusted kappa statistic (K*).

- *Content Validity Ratio* (*CVR*): This index was computed in accordance with the procedures outlined in Table 2.
- *Content Validity Index* (*CVI*): The Modified Kappa Statistic (K*) was utilized for this calculation, as detailed in Table 3 (Polit and Yang 2016; Polit and Beck 2017; Polit et al. 2007).

**TABLE 2:** Steps for calculating the CVR in the present study.

Step	Step description
1	Providing detailed explanations regarding the objectives of the instrument and the operational definitions.
2	Requesting 10 experts (faculty members with experience and research background in nursing management and instrument development) to assess the necessity of each item using a three‐point Likert scale: “essential,” “useful but not essential,” and “not necessary.”
3	Calculating content validity ratio (CVR) using the following formula: $\mathrm{CVR}=\frac{n_{e}-\left(\frac{N}{2}\right)}{\frac{N}{2}}$ In this formula, *ne* represents the number of experts who selected “essential,” and *N* denotes the total number of experts.

*Note:* After calculating the CVR, a minimum acceptable value of 0.62 was determined at a 95% confidence level, based on Lawshe's table and the number of experts. Accordingly, items with a CVR less than 0.62 were eliminated. The remaining items were then evaluated for content validity index (CVI) using the K* statistic.

**TABLE 3:** Steps for calculating K* in the present study.

Step	Description of step
1	Initially, the Content Validity Index for each item (I‐CVI) was calculated using the conventional method based on the formula proposed by Waltz and Bausell. For this purpose, a panel of experts (10 university professors with sufficient experience in instrument development, management and nursing) evaluated each item for “relevance” using a four‐point Likert scale. Each item was rated from 1 (“not relevant”) to 4 (“highly relevant”). $\mathrm{CVI}=\frac{\left(\text{number of raters giving}\;a\;{\text{rating of}}^{'}3^{'}\;{\text{or}}^{'}4^{'}\right)}{\text{total number of raters}}$
2	The next step involved calculating the probability of chance agreement (Pc), which was computed using the following formula: $P_{C}=\left[\frac{N!}{A!\left(N-A\right)!}\right]\times 0.5^{N}$ In this formula, A represents the number of experts who rated the item as 3 or 4, and N denotes the total number of experts.
3	In the final step, the modified kappa statistic (K*) was calculated using the following formula: $K*=\frac{I-\mathrm{CVI}-P_{c}}{1-P_{c}}$

*Note:* According to Polit, the interpretation of K* is as follows: a value between 0.40 and 0.59 is considered weak, between 0.60 and 0.74 is good and above 0.74 is excellent (Polit and Yang 2016). Based on this criterion, in the present study, items with a K* greater than 0.74 were considered highly relevant and excellent.

### 2.6 Initial Reliability Assessment

During the pilot testing phase, preliminary item analysis and initial internal consistency were evaluated using Cronbach's alpha. The loop method and the correlation between each individual item and the total score were employed. Furthermore, the change in the alpha coefficient was examined upon the hypothetical deletion of each item. Notably, an item was considered for removal if its correlation with the total score was less than 0.30 (Polit and Yang 2016).

### 2.7 Determining Construct Validity

To determine construct validity, exploratory factor analysis was utilized. The instrument comprised a demographic section and the main scale items, which were rated on a five‐point Likert scale (always/often/sometimes/rarely/never). The inclusion criteria required participants to be currently employed in a hospital setting with at least six months of clinical experience and active involvement in direct patient care. A final sample size of 220 was enrolled, sufficiently exceeding the rule‐of‐thumb minimum requirement of 200 to account for potential attrition (Munro 2012). Exclusion criteria comprised careless or indifferent responding (identified by a response standard deviation of less than 0.2) and the presence of univariate or multivariate outliers.

Initially, Bartlett's test of sphericity and the Kaiser‐Meyer‐Olkin (KMO) measure of sampling adequacy were employed to assess the suitability of the sample for factor extraction. A KMO index value exceeding 0.5 was considered acceptable (Polit and Yang 2016); however, values greater than 0.7 were deemed ideal, with 0.7 representing the minimum acceptable value for this index in the present study (Polit and Yang 2016). The number of factors to be extracted was determined based on the eigenvalues and the scree plot.

Subsequently, the final decision regarding the number of factors was made based on parallel analysis. Considering the eigenvalues, factors with an eigenvalue greater than 1 were examined, while factors with an eigenvalue less than 1 were disregarded (Polit and Yang 2016). The 95% confidence interval for each eigenvalue in the present study was calculated according to the following formula (Larsen and Warne 2010):

$$l_{i}\pm z^{*}\left(\sqrt{\frac{{2l}_{i}^{2}}{n}}\right)$$

where *l*_*i*_ is the eigenvalue and *n* is the sample size. For a 95% confidence interval, *z* was 1.96. An eigenvalue equal to or greater than 1 could be determined with 95% confidence using the confidence interval for each eigenvalue.

Based on the simulated scree plot, the number of extracted factors was determined by identifying factors whose observed eigenvalues exceeded the 95th percentile of the simulated data (O'connor 2000; Finch et al. 2016). Furthermore, to determine the minimum acceptable factor loading for retaining each item within a factor, the formula CV = 5.152 ÷ √(*n* − 2) was utilized with a 99% confidence level, where n represents the sample size (Norman and Streiner 2014). Consequently, a value of approximately 0.35 was calculated as the minimum acceptable factor loading.

In addition to the classical exploratory approach, Exploratory Graph Analysis (EGA) was conducted to evaluate the construct validity and dimensionality of the instrument from a network psychometrics perspective. EGA conceptualizes psychological constructs as a network of interacting items, estimating the number of dimensions without the rotational subjectivity inherent in traditional factor analysis. The network was estimated utilizing the cor_auto function to account for the ordinal nature of the data. Prior to structure estimation, a Unique Variable Analysis (UVA) was conducted to check for item redundancy and local dependence using the weighted topological overlap (wTO) metric. Furthermore, the structural stability of the dimensions and the stability of individual items within their respective communities were evaluated using the bootEGA function with 500 parametric resampling iterations. Model fit was evaluated using the Total Entropy Fit Index (TEFI), and network loadings were computed to determine the proportional contribution of each item to its assigned dimension.

To further investigate the unidimensionality and hierarchical properties of the instrument, Non‐parametric Item Response Theory (IRT) was applied using Mokken Scale Analysis (MSA). MSA evaluates the scalability of the items using Loevinger's scalability coefficient (H). An item scalability coefficient (Hi) of at least 0.30 is required to retain an item within the scale. The overall scale quality is classified as weak if 0.30 ≤ H < 0.40, moderate if 0.40 ≤ H < 0.50 and strong if H ≥ 0.50.

Furthermore, to empirically evaluate the replicability of the factor structure and ensure the absolute adequacy of the sample size, a Monte Carlo simulation was conducted. The simulation generated 1000 random datasets based on the empirical correlation matrix of the final 12 items. For each simulated dataset, a Principal Axis Factoring (PAF) extraction for a unidimensional structure was performed without rotation. The structural stability across simulations was evaluated using Tucker's Congruence Coefficient (CC). A CC value of ≥ 0.90 is widely recognized as an indicator of a highly similar and stable factor structure. Statistical power was calculated as the proportion of simulated samples that successfully achieved a CC ≥ 0.90.

### 2.8 Identification of Indifferent Respondents

To identify and remove indifferent respondents, the standard deviation of each participant's responses was utilized. Consequently, using Microsoft Excel 2007 and its built‐in functions, respondents with a standard deviation of less than 0.2 were excluded from the study.

### 2.9 Examination of Normality and Univariate and Multivariate Outliers

The normality of the data was assessed based on skewness (±3) and kurtosis (±7) indices. Box plots were employed to identify univariate outliers; potential cases for removal from the dataset were those identified as outliers across the majority of variables. To detect multivariate outliers, the Mahalanobis d‐squared test was conducted using Amos version 25. Cases with a significance level of *p* < 0.001 were considered potential multivariate outliers for removal.

### 2.10 Final Reliability

Internal consistency was assessed using Cronbach's alpha, and temporal stability was examined using the test–retest method.

#### 2.10.1 Cronbach's Alpha

In this study, a Cronbach's alpha coefficient greater than 0.70 was considered acceptable (Taber 2018). Additionally, the average inter‐item correlation (AIC) and McDonald's omega (ω) were examined. McDonald's omega was calculated based on the standardized factor loadings and item uniqueness derived from the exploratory factor analysis to provide a more robust estimate of internal consistency compared to Cronbach's alpha. Consistent with Glen's statement, an AIC value between 0.15 and 0.50 was considered desirable (Glen 2018). For this study, an AIC value ranging from 0.15 to 0.50 was considered optimal, and an omega value exceeding 0.70 was deemed acceptable (Stone et al. 2015). Cronbach's alpha and AIC were calculated using data from 30 participating nurses, and McDonald's omega was computed based on the results of the exploratory factor analysis.

#### 2.10.2 Test–Retest Reliability

To ascertain temporal stability, the intraclass correlation coefficient (ICC) was calculated from the test–retest data using a two‐way mixed‐effects model based on absolute agreement. An ICC value of 0.75 or above was considered indicative of acceptable reliability (Koo and Li 2016). The specific sample size of 30 for this temporal phase was predetermined via power analysis (alpha = 0.05, power = 0.8) (Bujang and Baharum 2017).

The relative reliability of the instrument was solely determined by the intraclass correlation coefficient (Polit and Yang 2016). Additionally, the standard error of measurement (SEM) was estimated to ascertain the absolute reliability in this study. The SEM was calculated using the following formula:

$$\mathrm{SEM}={\mathrm{SD}}_{\text{Pooled}}\times \sqrt{\left(1-{\mathrm{ICC}}_{\text{agreement}}\right)}$$

The pooled standard deviation (SD_Pooled_) was calculated using the following formula:

$${\mathrm{SD}}_{\text{Pooled}}=\left(\mathrm{SD}1+\mathrm{SD}2\right)/2$$

### 2.11 Scoring the Final Instrument

The final Nursing Care Rationing Instrument consists of 12 items, which are rated on a five‐point Likert scale ranging from 1 to 5 (5 = always, 4 = often, 3 = sometimes, 2 = rarely, 1 = never). To calculate the overall nursing care rationing score, the responses for all 12 items are summed, yielding a total possible score ranging from 12 to 60. There are no reverse‐scored items in this tool. In this scoring system, higher total scores reflect a greater frequency and extent of nursing care rationing, whereas lower scores indicate that nursing care tasks are omitted or rationed less frequently.

### 2.12 Statistical Analysis

Data management and classical statistical analyses, including descriptive statistics, preliminary item analysis and Exploratory Factor Analysis (EFA) with Principal Axis Factoring (PAF) extraction, were executed using IBM SPSS Statistics version 27.0. Parallel analysis and simulated scree plots were generated using JASP software (version 0.14). Advanced modern psychometric evaluations were conducted in R software, utilizing the EGAnet package for Exploratory Graph Analysis (GLASSO estimation and Louvain community detection) and the mokken package for Non‐parametric Item Response Theory (Mokken Scale Analysis). Monte Carlo simulations for structural replicability were strictly programmed based on the empirical correlation matrix. Power analysis for the temporal stability sample size was determined utilizing PASS software (version 11.0.7).

### 2.13 Ethical Considerations

This study was conducted in accordance with the ethical principles of the Declaration of Helsinki. The research protocol was reviewed and approved by the Institutional Research Ethics Committee of Kermanshah University of Medical Sciences (Approval Code: IR.KUMS.REC.1403.137). Prior to data collection, the study's objectives, voluntary nature of participation and the right to withdraw at any stage without consequence were thoroughly explained to all participants. Written informed consent was obtained from all individuals prior to their involvement. To guarantee confidentiality and preserve anonymity, no personally identifiable information was collected and all completed paper instruments were managed using sealed envelopes and anonymous alphanumeric tracking codes.

## 3 Results

### 3.1 Development of Items and Design of the Initial Version of the Instrument

Using an inductive‐deductive approach, an initial pool of 78 items was generated. Subsequently, during a review session conducted by the research team, overlapping items were merged and irrelevant items were removed. This process resulted in a preliminary version of the instrument comprising 45 items.

### 3.2 Instrument Validation

#### 3.2.1 Face Validity

Quantitative assessment of face validity: In this study, 11 items received an impact score below 1.5 and were deemed unsuitable by respondents, necessitating revision (Table S1).

Qualitative assessment of face validity: Based on interviews with 10 nurses, the 11 items identified as needing modification in the quantitative face validity stage were revised accordingly (Table S2). During this phase, the wording of ambiguous items was refined to enhance clarity and ease of understanding for the instrument's users (nurses).

#### 3.2.2 Content Validity

Qualitative content validity: Following interviews with 10 faculty members and incorporating their corrective feedback, the phrasing of one item was revised and refined to enhance conceptual clarity (Table S3).

#### 3.2.3 Quantitative Content Validity

- *Content Validity Ratio* (*CVR*): After implementing the qualitative content validity revisions, the instrument entered the CVR evaluation phase. At this stage, 23 items were removed due to CVR scores below 0.62, resulting in a 22‐item instrument prepared for Content Validity Index (CVI) assessment (Table S4).
- *Content Validity Index* (*CVI*): Based on the K* statistic, no additional items were eliminated in this phase; thus, the instrument, retaining 22 items, advanced to the initial reliability assessment stage (Table S5).

### 3.3 Initial Reliability Assessment

After establishing the content validity of the instrument, its initial reliability was examined. At this stage, item analysis was conducted to calculate Cronbach's alpha and to identify items that negatively affected the instrument's reliability. For this purpose, 30 nurses who were directly responsible for patient care in the hospital completed the instrument. The preliminary results indicated a Cronbach's alpha coefficient of 0.871. Subsequently, the correlation between each item and the total score was analysed, revealing that three items had correlations below 0.1 and were therefore removed. Following the removal of these items, the Cronbach's alpha coefficient increased to 0.942, indicating a desirable and acceptable level of reliability. Ultimately, the instrument, consisting of 19 items, proceeded to the construct validity phase.

### 3.4 Construct Validity Assessment

To assess construct validity, exploratory factor analysis (EFA) was conducted. A total of 220 nurses participated in the study. The adequacy of the sample was evaluated using the Kaiser‐Meyer‐Olkin (KMO) measure, which was estimated at 0.947, indicating excellent suitability for factor analysis. Bartlett's test of sphericity was also significant (*p* < 0.001), confirming the appropriateness of the data for factor extraction.

To determine the number of factors to extract, the scree plot was examined visually. It suggested that a single factor could adequately explain the underlying construct of the nursing care rationing instrument, as only the first factor had an eigenvalue exceeding the 95th percentile of eigenvalues derived from simulated data (Figure 1).

**FIGURE 1:**
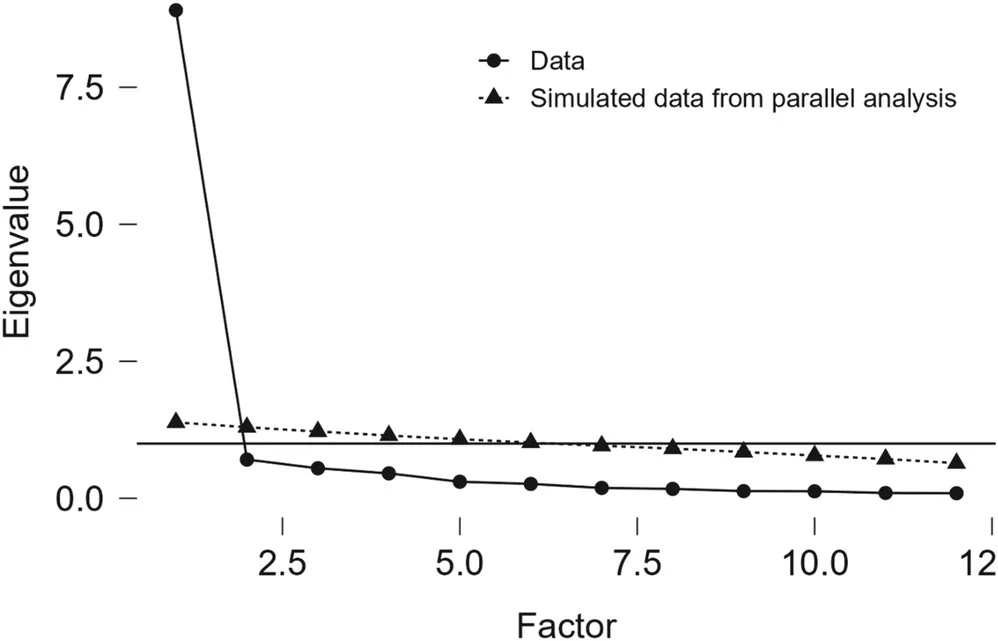
Scree plot to determine the number of tool factors.

Parallel analysis further supported this conclusion. As shown in Table 4, the observed eigenvalue for the first factor was numerically larger than the corresponding random eigenvalue, indicating that this factor was statistically significant and should be retained. Accordingly, only one factor was considered the precise number of factors to extract. Moreover, based on the 95% confidence interval, it can be confirmed with 95% confidence that the eigenvalue for the extracted factor is equal to or greater than one.

**TABLE 4:** Parallel analysis results based on observed and random eigenvalues.

	Real data factor eigenvalues (95% confidence interval)	Simulated data mean eigenvalues
Factor 1^a^	8.907 (8.057751–9.756249)	1.386
Factor 2	0.707 (0.63959–0.77441)	1.298
Factor 3	0.549 (0.496655–0.601345)	1.219
Factor 4	0.456 (0.412522–0.499478)	1.147
Factor 5	0.303 (0.27411–0.33189)	1.079
Factor 6	0.263 (0.237924 0.288076)	1.017

^a^ Factor should be retained.

Following the confirmation of multivariate normality assumptions via skewness and kurtosis indices, an initial Exploratory Factor Analysis (EFA) using Principal Axis Factoring (PAF) extraction was performed on the 19‐item pool. During the iterative analytical steps, seven items were systematically eliminated to refine the model. The criteria for removal included low extraction communalities (*h* ^2^ < 0.20), factor loadings below the a priori threshold of 0.30 or the presence of significant cross‐loadings across multiple dimensions in the preliminary stages. Given that the scree plot, parallel analysis and the refined item pool strongly supported a single‐factor solution, no rotation was applied to the final model. As detailed in Table 5, the final retained unidimensional model, consisting of 12 items, successfully explained 71.97% of the total variance (based on extraction sums of squared loadings), demonstrating a highly cohesive factor structure. The specific factor loadings (ranging from 0.743 to 0.918) and communalities (ranging from 0.552 to 0.842) for the final retained items are reported in Table 6.

**TABLE 5:** Eigenvalues and explained variance for the extracted factors.

Factor	Initial eigenvalues			Extraction sums of squared loadings		
	Total	% of variance	Cumulative %	Total	% of variance	Cumulative %
1	8.907	74.221	74.221	8.636	71.967	71.967
2	0.707	5.895	80.117			
3	0.549	4.574	84.690			
4	0.456	3.799	88.490			
5	0.303	2.527	91.016			
6	0.263	2.193	93.209			
7	0.190	1.587	94.796			
8	0.173	1.440	96.236			
9	0.131	1.096	97.331			
10	0.129	1.072	98.403			
11	0.098	0.817	99.220			
12	0.094	0.780	100.000			

*Note:* Extraction method: principal axis factoring.

**TABLE 6:** Extracted factor loadings and communalities.

Items	Factor 1	Communalities (*h* ^2^)
I am faced with a lack of time to provide complete care services to patients.	0.918	0.842
I should allocate more time for each patient's nursing care, but the large number of patients does not allow it.	0.911	0.830
To attend to all patients, I am forced to overlook some services.	0.886	0.785
I am forced to ration nursing care based on time and resource limitations.	0.870	0.756
I am forced to ration nursing care to distribute available resources fairly among patients.	0.869	0.755
Due to lack of time, I am forced to prioritize patient care.	0.863	0.745
To gain time to perform medical care, I overlook some education for the patient and their companion.	0.847	0.717
Despite overlooking some care, I still do not have the ability to fully attend to all my patients.	0.840	0.706
The number of patients assigned to me exceeds my ability to provide adequate care.	0.838	0.703
To attend to the patients under my care, I am forced to overlook some aspects of one patient's care to attend to another patient.	0.830	0.689
I am worried that I cannot fully perform all the care for my patients.	0.745	0.555
In providing patient care, due to lack of time, I entrust some care to the patient or their companion.	0.743	0.552

To robustly confirm the unidimensional structure derived from the factor analysis, a network psychometrics approach via EGA was applied to the final 12 items. Prior to extracting the network, the UVA redundancy check indicated moderate to large topological overlaps among specific item pairs (e.g., Q16–Q19 with wTO = 0.377, and Q7–Q8 with wTO = 0.306). Given their theoretical relevance and contribution to the core construct of care rationing, these items were retained for the network estimation.

The application of the EGA algorithm supported a unidimensional network structure (a single community) for the 12‐item instrument, consistent with the EFA solution (Figure 2). Dimension stability evaluated via bootEGA (n=500 resamples) indicated a median dimension of 1 (95% CI: 1–1; SE=0). Furthermore, item stability analysis demonstrated consistent replication rates (1.00 or 100%) across all 12 nodes, indicating that items consistently clustered within the single identified community throughout all bootstrap iterations. Network loadings, representing the strength of an item's association with the entire network, ranged from 0.285 to 0.621, further substantiating the items' strong integration into the singular construct.

**FIGURE 2:**
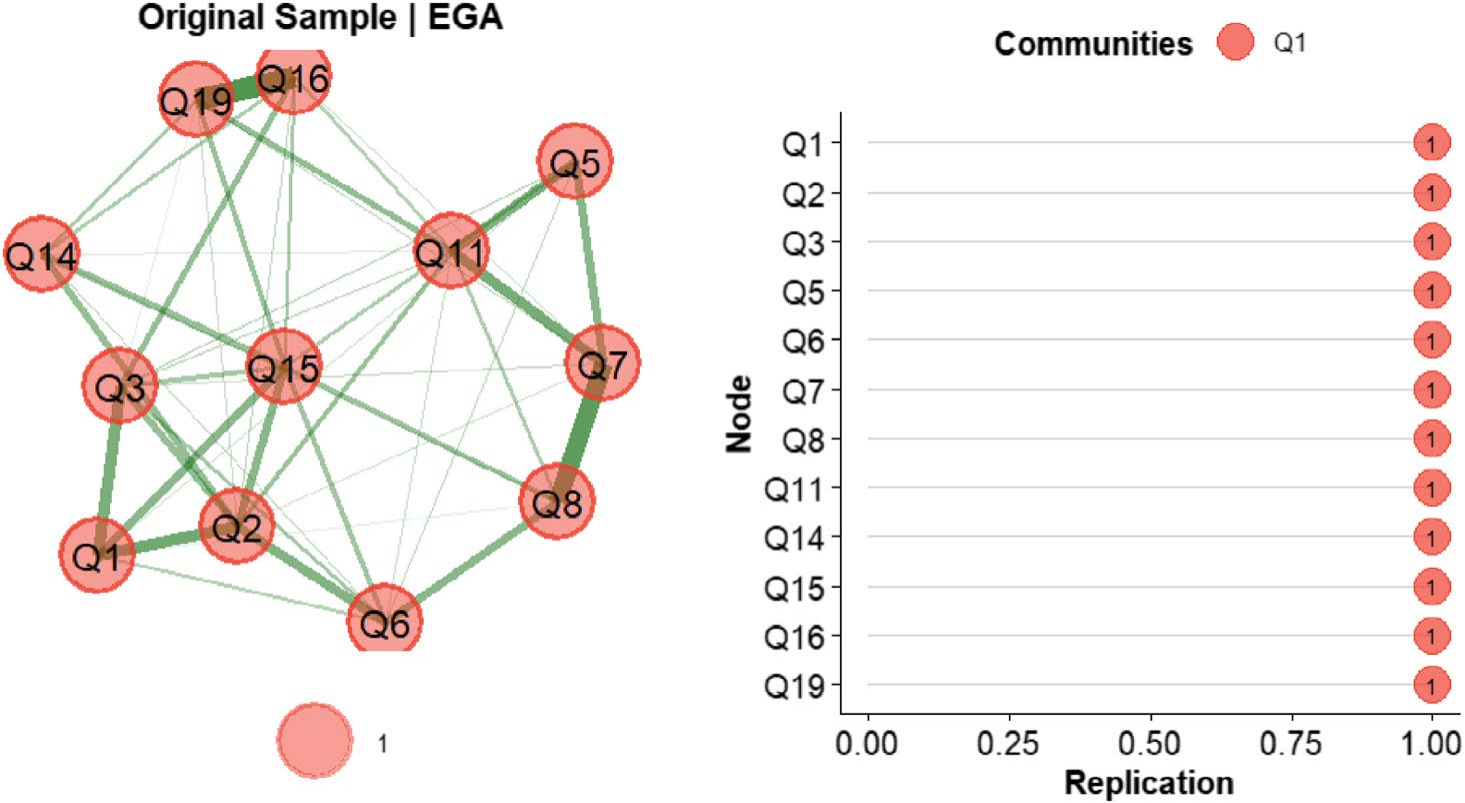
Network structure of the 12‐item Nursing Care Rationing Instrument estimated via Exploratory Graph Analysis (EGA). Nodes (circles) represent the individual items, and edges (connecting lines) illustrate the strength of the relationships (partial correlations) between them. The single colour applied to all nodes confirms the strictly unidimensional structure of the construct.

The Mokken Scale Analysis demonstrated excellent scalability for the final 12‐item instrument among the 220 participating nurses. The overall Loevinger's H coefficient was 0.762, indicating a “strong” unidimensional scale. Furthermore, the item‐level scalability coefficients (H_i_) ranged from 0.677 to 0.817 (Table 7). Since all item coefficients were substantially above the 0.30 threshold, it was confirmed that no items required removal, and the hierarchical structure of the instrument is highly robust.

**TABLE 7:** Item‐level scalability coefficients (H_i_) from Mokken Scale Analysis.

Item (node)	Scalability coefficient (Hi)	Scale quality evaluation
Q1	0.766	Strong
Q2	0.817	Strong
Q3	0.774	Strong
Q5	0.680	Strong
Q6	0.758	Strong
Q7	0.764	Strong
Q8	0.759	Strong
Q11	0.797	Strong
Q14	0.677	Strong
Q15	0.817	Strong
Q16	0.772	Strong
Q19	0.772	Strong

*Note:* Overall Loevinger's H = 0.762, indicating a strong unidimensional scale. According to Mokken's criteria, an H_i_ ≥ 0.50 is considered strong. All items exceeded the minimum retention threshold of 0.30.

The Monte Carlo simulation (1000 replications) strongly supported the stability of the unidimensional construct and the adequacy of the sample size (*n* = 220). The mean Tucker's Congruence Coefficient across all 1000 simulated datasets was 1.00, representing a perfect structural match with the empirical model. Consequently, the statistical power to recover the factor structure was 100%, indicating that every single simulated sample successfully achieved a Congruence Coefficient of ≥ 0.90. This confirms that the extracted 12‐item structure is highly replicable.

### 3.5 Determining Final Reliability

#### 3.5.1 Internal Consistency Determination

In this study, to assess the internal consistency of the instrument, Cronbach's alpha coefficient was calculated. Given that the Cronbach's alpha index can artificially increase with larger sample sizes and a greater number of items (Selk‐Ghaffari et al. 2022), McDonald's omega (ω) was also reported alongside Cronbach's alpha. Accordingly, the overall Cronbach's alpha coefficient for the instrument was 0.953, and McDonald's omega was 0.968. Additionally, the AIC coefficient was reported as 0.633.

#### 3.5.2 Stability Assessment via Test–Retest Method

To evaluate the stability of the instrument, a test–retest method was employed. The instrument was initially completed by 30 clinical nurses and then re‐administered to the same participants after a two‐week interval. The intraclass correlation coefficient (ICC) for the entire instrument between the two measurements was 0.969, with a 95% confidence interval ranging from 0.953 to 0.985.

## 4 Discussion

In the present study, the process of designing and validating the instrument resulted in the development of a nursing care rationing tool comprising 12 items. The items in this tool address factors such as the number of patients under care, time constraints, equitable distribution of resources among patients, shortage of care facilities, comprehensive attention to all patients, omission of patient and family education, delegation of certain care tasks to patients and their companions and prioritization of therapeutic care.

A comparison between the developed instrument and the PRINCA tool, designed by Dominika Kalánková and colleagues, reveals that some items in Kalánková's tool partially overlap conceptually with those in the present instrument (Kalánková et al. 2020). For example, the item “To gain time to perform medical care, I overlook some education for the patient and their companion.” in the current tool inversely corresponds to the item “Providing the amount of teaching for the patient or his/her family” in PRINCA. Additionally, the item “Provide adequate supervision of or follow‐up on delegated activities” in PRINCA is somewhat related to the item “I should allocate more time for each patient's nursing care, but the large number of patients does not allow it.” in the present tool (Kalánková et al. 2020).

Overall, although some items in the current instrument partially align with those in PRINCA, the latter does not precisely capture the specific items developed in this study.

The initial step in validating the examined instrument involved establishing its face validity through the feedback of its users. Specifically, each item of the instrument was evaluated by nurses in terms of clarity, lack of ambiguity and appropriate phrasing. A review of previous studies on the rationing of nursing care revealed no evidence of face validity assessment using either quantitative or qualitative methods with the precision required for our tool. This gap is notable, especially considering Allen and Baruch Nevo's assertion that face validity holds such critical importance that it cannot be substituted by other stages of construct validation (Nevo 1985; Allen et al. 2023; Sidik 2018). According to Ghaffari et al., a comprehensive approach to instrument validation—including face validity—can result in a more accurate and reliable tool (Selk‐Ghaffari et al. 2022). Ronald Holden further emphasizes that neglecting face validity ultimately produces an instrument that causes dissatisfaction and misunderstanding of the questionnaire's core concepts among end users, thereby reducing the survey's efficacy and quality (Holden 2018).

During the face validity phase, difficult or ambiguous words and phrases are reviewed to minimize misinterpretation and items that do not align well with the instrument's dimensions are identified. Consequently, end users provide more constructive feedback on the tool (Holden 2018; Connell et al. 2018). In this study, both qualitative and quantitative methods were employed to determine face validity. In the qualitative approach, items were revised to be more fluent and comprehensible for the future users—namely, nurses—and ambiguities were resolved. In the quantitative approach, an item's impact score exceeding 1.5, as rated by respondents, was considered a criterion for item approval. According to Ronald Holden, establishing face validity through item revision and review reduces the gap between the instrument and its end users (Holden 2018).

The content validity of the instrument under consideration was assessed using both quantitative and qualitative methods, according to a panel of experts in instrument design and psychometrics. This stage ensured the instrument's ability to measure the concept of nursing care rationing. A review of prior research conducted by Schubert et al. on the development of a nursing care rationing instrument did not reveal any report of content validity assessment using quantitative and qualitative methods (Schubert et al. 2008). In studies conducted by Izabela Uchmanowicz et al. titled “(Validation of Polish version of the Basel Extent of Rationing of Nursing Care revised questionnaire),” which aligns with the topic of our research, the Basel questionnaire was translated into Polish, and its content validity was examined. Although Uchmanowicz's study did address content validity, this assessment was solely performed by a number of nurses undergoing specialized training in internal nursing care at the Center for Postgraduate Medical Education (Uchmanowicz et al. 2019). In contrast, the content validity in the present study was evaluated and confirmed by nursing faculty members. This oversight in the rigorous assessment of content validity is significant, as the proper determination of content validity influences the results of factor analysis. Furthermore, an inadequate assessment of content validity can compromise the reliability of the instrument (Rubio et al. 2003); consequently, the reliability of Uchmanowicz's instrument may have been affected by this issue.

To determine construct validity, exploratory factor analysis (EFA) was employed. To assess sampling adequacy, the Kaiser‐Meyer‐Olkin (KMO) index was calculated to be 0.947, a value considered excellent and acceptable. Furthermore, Bartlett's test of sphericity was found to be statistically significant. To determine the number of factors to extract, in addition to classical criteria, modern approaches such as parallel analysis were utilized as a precise method for identifying the number of items. According to Silverstein's assertion regarding the comparison between the Kaiser criterion and parallel analysis, parallel analysis demonstrates greater accuracy than the Kaiser criterion (Silverstein 1990). Following the execution of the exploratory factor analysis, the final instrument comprised 12 items, explaining 71.97% of the total variance. This level of explained variance is considered acceptable based on the 50% criterion. Ingrid Andersson et al. conducted a study titled ‘Validation of the Basel Extent of Rationing of Nursing Care for Nursing Homes and Home Care, a Swedish version’; although this study also employed exploratory factor analysis, it resulted in the explanation of 46.942% of the total variance (Andersson et al. 2023), a statistically significant difference compared to the total variance explained by the present instrument. Moreover, Anderson's research is specifically applicable to nursing homes and home care settings, whereas the current study has no such limitations.

The robustness of this unidimensionality was further corroborated by the application of modern network psychometrics via Exploratory Graph Analysis (EGA). The network approach provided a significant methodological advantage over traditional exploratory techniques by visually and mathematically modelling the direct interactions among the rationing behaviours. The high structural stability demonstrated by the bootstrap analysis—achieving a 100% replication rate of the single dimension across 500 iterations‐indicates that the 12‐item construct is highly resilient to sample fluctuations. While local dependence was observed among a few item pairs (e.g., items related to time constraints and educational omissions), this shared variance is likely due to semantic similarities in specific clinical rationing scenarios and did not compromise the model. The overarching perfect item stability within the single community underscores that the instrument measures a singular, highly cohesive construct. This solidifies the tool's reliability, offering healthcare managers a precise and modern metric for evaluating nursing care rationing that is unencumbered by the multidimensional ambiguities seen in earlier instruments.

The structural robustness of the newly developed instrument was further reinforced by the results of the Mokken Scale Analysis (MSA). As a non‐parametric Item Response Theory approach, MSA confirmed a remarkably strong hierarchical and unidimensional structure (Loevinger's H = 0.762). This strong scalability implies a cumulative property within the instrument; essentially, nurses who endorse items representing higher levels or more severe forms of nursing care rationing are consistently likely to endorse items representing lower‐level rationing. This multifaceted evaluation (combining factor analysis with non‐parametric Item Response Theory and network psychometrics) provides robust empirical evidence for the instrument's structural integrity, setting it apart from previously developed tools and ensuring its theoretical soundness in clinical assessments.

In this study, Cronbach's alpha coefficient was calculated to determine the internal consistency of the instrument. Furthermore, considering that the Cronbach's alpha index can spuriously increase with larger sample sizes and a greater number of items (Polit and Yang 2016), McDonald's omega (ω) was reported in addition to Cronbach's alpha. Accordingly, the Cronbach's alpha coefficient for the entire instrument was reported as 0.953, and McDonald's omega for the entire instrument was 0.968. Additionally, the AIC coefficient in this study was 0.633. While the classical reliability indices (alpha and omega > 0.95) represent excellent internal consistency, the AIC value exceeding the theoretically desirable upper limit of 0.50 suggests a degree of item redundancy. This interpretation aligns with the moderate local dependence observed during the network redundancy check (UVA). In clinical practice, such semantic overlap is expected because rationing behaviours are highly interconnected; a nurse compelled to ration one aspect of care due to time constraints is likely rationing other closely related tasks simultaneously. In the research by Terry Jones titled “Validation of the Perceived Implicit Rationing of Nursing Care (PIRNCA) Instrument” (Jones 2014), no evidence of the test–retest method was found. For a more comprehensive evaluation of the instrument's reliability, similar to the present study, supplementary methods such as test–retest reliability should also be employed. This approach can provide a more precise insight into the stability and reliability of the instrument over time. For instance, utilizing the test–retest method can demonstrate the extent of response stability across different time intervals, thereby providing greater confidence in the instrument's dependability in real‐world conditions. Moreover, combining this method with Cronbach's alpha and McDonald's omega coefficients allows for a multifaceted analysis of reliability, assisting researchers in evaluating the instrument from various perspectives. Ultimately, this comprehensive approach can lead to the enhancement of the instrument's quality and increase its accuracy in measuring the rationing of nursing care.

### 4.1 Study Limitations and Future Research

While this study presents a robust psychometric evaluation, several limitations must be acknowledged. First, conducting the study within a single geographic region may limit the broad generalizability of the findings. Second, reliance on self‐reported data may introduce response bias, as nurses might underreport rationing behaviours due to social desirability. Importantly, regarding the structural validation, while a traditional Confirmatory Factor Analysis (CFA) on an independent sample is typically recommended as a subsequent step, we proactively addressed the stability and replicability of the instrument through advanced computational methodologies. The application of Exploratory Graph Analysis (EGA) with bootstrap resampling, Non‐parametric Item Response Theory (Mokken Scale Analysis) and a Monte Carlo simulation (1000 replications) rigorously compensated for the absence of CFA in this initial developmental phase. Specifically, the Monte Carlo simulation demonstrated a perfect structural replication rate, providing compelling evidence of the scale's stability that parallels cross‐validation techniques. Nevertheless, future research should aim to conduct a formal CFA in an independent, multi‐center sample to further confirm this unidimensional structure. Additionally, future studies should evaluate criterion‐related, convergent and discriminant validity across diverse healthcare settings and cultural populations.

### 4.2 Implications for Nursing Management

The clinical relevance of this newly developed instrument lies in its practical application for healthcare managers. By providing a reliable, context‐specific metric for nursing care rationing, nursing directors can empirically identify units facing severe resource constraints. This facilitates data‐driven decision‐making for equitable staff allocation, targeted interventions to reduce workload‐induced burnout, and ultimately, the enhancement of patient safety protocols within Iranian hospitals.

## 5 Conclusion

In the present study, a 12‐item instrument with a single dimension was developed and demonstrated desirable psychometric properties. All items of the developed instrument underwent thorough psychometric evaluation and exhibited appropriate validity and reliability characteristics. Consequently, this study provides preliminary evidence for a newly developed instrument that, possessing satisfactory validity and reliability, enables the measurement of the extent of nursing care rationing in the Iranian context. Specifically, higher scores indicate a greater degree of rationing, whereas lower scores suggest less rationing by nurses. Given its preliminary nature, further validation – including independent Confirmatory Factor Analysis and cross‐cultural testing‐ is recommended to solidify its broader applicability.

## Author Contributions

**Mohammad Mehdi Mohammadi:** conceptualization, investigation, funding acquisition, writing – original draft, writing – review and editing, validation, visualization, methodology, software, project administration, formal analysis, resources, supervision, data curation. **Mohammad Mohammadi:** investigation, funding acquisition, writing – original draft, methodology, validation, visualization, writing – review and editing, project administration, formal analysis, software, data curation, supervision, resources, conceptualization. **Sara Bakhtiyari:** conceptualization, investigation, funding acquisition, writing – original draft, methodology, validation, visualization, writing – review and editing, project administration, formal analysis, software, data curation, supervision, resources.

## Funding

The present study was financially supported by Kermanshah University of Medical Sciences, Kermanshah, Iran (no. 4030244). We extend our sincere gratitude to the Student Research Committee of Kermanshah University of Medical Sciences for their financial contribution, which was instrumental in the successful completion of this research. The funders had no role in study design, data collection and analysis, decision to publish or preparation of the manuscript.

## Ethics Statement

This study was ethically approved by the ethics committee of Kermanshah University of Medical Sciences (IR.KUMS.REC.1403.137). The researcher obtained the written informed consent of each participant. Participants were assured that they had the right to withdraw from the study at any stage.

## Consent

The authors have nothing to report.

## Conflicts of Interest

The authors declare no conflicts of interest.

## Supporting information


**Table S1:** The item impact score in the quantitative face validity.

**Table S2:** Revised items in the qualitative Face validity phase.

**Table S3:** Revised items in the qualitative content validity stage.

**Table S4:** CVR results of items in the quantitative content validity.

**Table S5:** I‐CVI results based on K*.

## Data Availability

The data that support the findings of this study are available on request from the corresponding author. The data are not publicly available due to privacy or ethical restrictions.
