# Supplementary material for: Design and Psychometric Evaluation of a Nursing Care Rationing Instrument
Source: Nurs Open. 2026 Oct 16;13(10):e70933. doi: 10.1002/nop2.70933 (PMC13675515; doi:10.1002/nop2.70933)
Supplement: Supplementary file 1 [file NOP2-13-e70933-s001.doc]

| **Appendix Table S4.** CVR results of items in the quantitative content validity | | | |
| --- | --- | --- | --- |
| **#** | **Items** | **CVR** | **Results** |
| 1 | The number of patients assigned to me exceeds my ability to provide adequate care | 1 | Confirmed |
| 2 | Due to staff shortage, my workload has increased. | 0.6 | Deleted |
| 3 | I am faced with a lack of time to provide complete care services to patients. | 0.8 | Confirmed |
| 4 | The increase in my shifts has caused me to lack the energy to perform complete care. | 0.6 | Deleted |
| 5 | Due to lack of time, I am forced to prioritize patient care. | 0.8 | Confirmed |
| 6 | The increase in the number of patients under my care has caused me difficulty in providing complete care. | 0.6 | Deleted |
| 7 | Low salary has caused me not to provide complete care for patients. | 0.2 | Deleted |
| 8 | In providing patient care, I am forced to ask for help from my colleagues. | 0.8 | Confirmed |
| 9 | In providing patient care, due to lack of time, I entrust some care to the patient or their companion. | 0.8 | Confirmed |
| 10 | I have enough time to provide complete education to the patient or their companion. | 0.8 | Confirmed |
| 11 | Despite overlooking some care, I still do not have the ability to fully attend to all my patients. | 0.8 | Confirmed |
| 12 | To gain time to perform medical care, I overlook some education for the patient and their companion. | 1 | Confirmed |
| 13 | To attend to the patients under my care, I am forced to overlook some aspects of one patient's care to attend to another patient. | 0.8 | Confirmed |
| 14 | In performing medical procedures, I carry out even the most minor doctor's orders. | 0.4 | Deleted |
| 15 | My flexibility has prevented me from getting stressed about attending to a large number of patients. | 0 | Deleted |
| 16 | Due to lack of facilities, I overlook some care. | 0.4 | Deleted |
| 17 | After the end of my shift, I realize that I have forgotten to perform some care. | 0,8 | Confirmed |
| 18 | The high volume of responsibility has caused me to overlook some care. | 0 | Deleted |
| 19 | The crowdedness of the workplace has caused me to eliminate some care. | 0.4 | Deleted |
| 20 | Writing nursing reports takes away my opportunity to perform some care. | 0.6 | Deleted |
| 21 | Due to the large number of patients, I have little time to establish proper communication with patients. | 0.2 | Deleted |
| 22 | I feel guilty for overlooking some services. | 0.8 | Confirmed |
| 23 | Due to not having enough rest time, I eliminate some aspects of patient care so that I can get some rest. | 0 | Deleted |
| 24 | Family concerns have caused me to forget to perform some patient care. | 0 | Deleted |
| 25 | Due to lack of time, I cannot answer the questions of the patient or their companion. | 0.6 | Deleted |
| 26 | To attend to all patients, I am forced to overlook some services. | 0.8 | Confirmed |
| 27 | My nursing shifts each month are standard and proportionate to my ability level. | 0.8 | Confirmed |
| 28 | Due to the large number of patients under my care, I am forced to ration my time to attend to all patients. | 0.4 | Deleted |
| 29 | Due to a shortage of resources, I am forced to ration therapeutic care for patients | 0.4 | Deleted |
| 30 | Rationing nursing care contributes to an increase in my care-related errors. | 0.8 | Confirmed |
| 31 | Due to my inability to manage the available resources, I am compelled to eliminate some of the care services. | 0.4 | Deleted |
| 32 | Due to the large number of my patients, I am forced to ration the amount of time I am present at the patient's bedside. | 0.4 | Deleted |
| 33 | Due to the high volume of care my patients require, despite their small number, I cannot be available to some patients for an extended period. | 0.4 | Deleted |
| 34 | Due to heavy workload, I cannot adequately follow up on the condition of my patients. | 0.6 | Deleted |
| 35 | I cannot balance between patients who need more care and other patients. | 0.8 | Confirmed |
| 36 | I am worried that I cannot fully perform all the care for my patients. | 0.8 | Confirmed |
| 37 | I should allocate more time for each patient's nursing care, but the large number of patients does not allow it. | 0.8 | Confirmed |
| 38 | My patients' psychosocial needs are overlooked due to lack of time. | 0.6 | Deleted |
| 39 | I am forced to ration nursing care to distribute available resources fairly among patients. | 0.8 | Confirmed |
| 40 | I have to make decisions based on the needs of the patients. | 0.8 | Confirmed |
| 41 | Due to the lack of assistance in providing care, I cannot provide complete medical care to patients. | 0.2 | Deleted |
| 42 | Due to lack of skill in performing care correctly, I am forced to overlook some patient care. | 0.8 | Confirmed |
| 43 | I am forced to ration nursing care based on time and resource limitations. | 0.8 | Confirmed |
| 44 | In performing my nursing duties, I consider organizational limitations (including resources and legal frameworks). | 0.6 | Deleted |
| 45 | While performing care, I feel that I have enough time to perform all the care for the patients. | 0.8 | Confirmed |
