# Supplementary material for: Design and Psychometric Evaluation of a Nursing Care Rationing Instrument
Source: Nurs Open. 2026 Oct 16;13(10):e70933. doi: 10.1002/nop2.70933 (PMC13675515; doi:10.1002/nop2.70933)
Supplement: Supplementary file 2 [file NOP2-13-e70933-s002.doc]

**Appendix Table S1.** The item impact score in the quantitative face validity

| **# Items** | Items | Suitability | | Item Impact Score | Result |
| --- | --- | --- | --- | --- | --- |
|  |  | Mean | Frequency |  |  |
| 1 | The number of patients under my care is more than my capacity. | 3.7 | 0.7 | 2.59 | Confirmed |
| 2 | Due to staff shortage, my workload has increased. | 3.9 | 0.7 | 2.73 | Confirmed |
| 3 | I face a lack of time in providing complete care services to patients. | 3.2 | 0.4 | 1.28 | Needs to be revised |
| 4 | The increase in my shifts has caused me to lack the energy to perform complete care. | 4 | 0.7 | 2.8 | Confirmed |
| 5 | Due to lack of time, I am forced to prioritize patient care. | 3.2 | 0.5 | 1.6 | Confirmed |
| 6 | The increase in the number of patients under my care has caused me difficulty in providing complete care. | 3.6 | 0.5 | 1.8 | Confirmed |
| 7 | Low salary has caused me not to provide complete care for patients. | 4 | 0.8 | 3.2 | Confirmed |
| 8 | In providing patient care, I am forced to ask for help from my colleagues. | 4 | 0.8 | 3.2 | Confirmed |
| 9 | In providing patient care, due to lack of time, I entrust some care to the patient or their companion. | 3.5 | 0.5 | 1.75 | Confirmed |
| 10 | I have enough time to provide complete education to the patient or their companion. | 3.2 | 0.5 | 1.6 | Confirmed |
| 11 | Despite overlooking some care, I still do not have the ability to fully attend to all my patients. | 3.2 | 0.5 | 1.6 | Confirmed |
| 12 | To gain time to perform medical care, I overlook some education for the patient and their companion. | 3.6 | 0.6 | 2.16 | Confirmed |
| 13 | To attend to all the patients under my care, I am forced to overlook some care of one patient to attend to another patient. | 2.5 | 0.2 | 0.5 | Needs to be revised |
| 14 | In performing medical procedures, I carry out even the most minor doctor's orders. | 4.2 | 0.9 | 3.78 | Confirmed |
| 15 | My flexibility has prevented me from getting stressed about attending to a large number of patients. | 3.6 | 0.5 | 1.8 | Confirmed |
| 16 | Due to lack of facilities, I overlook some care. | 4.1 | 0.6 | 2.46 | Confirmed |
| 17 | After the end of my shift, I realize that I have forgotten to perform some care. | 3.8 | 0.6 | 2.28 | Confirmed |
| 18 | The high volume of responsibility has caused me to overlook some care. | 3.7 | 0.6 | 2.22 | Confirmed |
| 19 | The crowdedness of the workplace has caused me to eliminate some care. | 3.3 | 0.5 | 1.65 | Confirmed |
| 20 | Writing nursing reports takes away my opportunity to perform some care. | 3.6 | 0.5 | 1.8 | Confirmed |
| 21 | Due to the large number of patients, I have little time to establish proper communication with patients. | 3.8 | 0.6 | 2.28 | Confirmed |
| 22 | I feel guilty for overlooking some services. | 3.8 | 0.7 | 2.66 | Confirmed |
| 23 | Due to lack of rest time, I eliminate some patient care so that I can rest a little. | 2.9 | 0.3 | 0.87 | Needs to be revised |
| 24 | Family concerns have caused me to forget to perform some patient care. | 3.7 | 0.6 | 2.22 | Confirmed |
| 25 | Due to lack of time, I cannot answer the questions of the patient or their companion. | 3.6 | 0.6 | 2.16 | Confirmed |
| 26 | To attend to all patients, I am forced to overlook some services. | 3.1 | 0.5 | 1.55 | Confirmed |
| 27 | My nursing shifts each month are standard and proportionate to my ability level. | 3.3 | 0.5 | 1.65 | Confirmed |
| 28 | Due to the large number of patients under my care, I am forced to ration my time to attend to all patients. | 3.2 | 0.5 | 1.6 | Confirmed |
| 29 | Due to lack of resources, I am forced to ration medical care for patients. | 2.6 | 0.2 | 0.52 | Needs to be revised |
| 30 | Rationing nursing care is effective in increasing my errors in care. | 2.8 | 0.2 | 0.56 | Needs to be revised |
| 31 | Due to my inability to manage available resources, I am forced to eliminate some care. | 2.9 | 0.4 | 1.16 | Needs to be revised |
| 32 | Due to the large number of my patients, I am forced to ration the amount of time I am present at the patient's bedside. | 3.2 | 0.5 | 1.6 | Confirmed |
| 33 | Despite the small number of my patients, due to the heavy care required for each patient, I am forced to have limited time available for some patients. | 2.9 | 0.3 | 0.87 | Needs to be revised |
| 34 | Due to heavy workload, I cannot adequately follow up on the condition of my patients. | 3.8 | 0.7 | 2.66 | Confirmed |
| 35 | I cannot balance between patients who need more care and other patients. | 3.3 | 0.5 | 1.65 | Confirmed |
| 36 | I am worried that I cannot fully perform all the care for my patients. | 3.9 | 0.7 | 2.73 | Confirmed |
| 37 | I should allocate more time for each patient's nursing care, but the large number of patients does not allow it. | 3.8 | 0.8 | 3.04 | Confirmed |
| 38 | My patients' psychosocial needs are overlooked due to lack of time. | 3.5 | 0.5 | 1.75 | Confirmed |
| 39 | I am forced to ration my nursing care based on the fair distribution of resources among patients. | 2.6 | 0.3 | 0.78 | Needs to be revised |
| 40 | I have to make decisions based on an accurate assessment of patients' needs. | 3.1 | 0.4 | 1.24 | Needs to be revised |
| 41 | I feel that due to a shortage of support staff, I cannot provide complete nursing care to patients. | 3 | 0.2 | 0.6 | Needs to be revised |
| 42 | Due to lack of skill in performing care correctly, I am forced to overlook some patient care. | 3.2 | 0.5 | 1.6 | Confirmed |
| 43 | I am forced to ration nursing care based on time and resource limitations. | 3.5 | 0.6 | 2.1 | Confirmed |
| 44 | In my nursing decisions, I consider organizational limitations. | 3.3 | 0.4 | 1.32 | Needs to be revised |
| 45 | While performing care, I feel that I have enough time to perform all the care for the patients. | 3.5 | 0.7 | 2.45 | Confirmed |
