# Supplementary material for: Design and Psychometric Evaluation of a Nursing Care Rationing Instrument
Source: Nurs Open. 2026 Oct 16;13(10):e70933. doi: 10.1002/nop2.70933 (PMC13675515; doi:10.1002/nop2.70933)
Supplement: Supplementary file 3 [file NOP2-13-e70933-s003.doc]

| **Appendix Table S5**. I-CVI results based on K* | | | | | |
| --- | --- | --- | --- | --- | --- |
| # | Items | I-CVI | Pc | K* | Results |
| 1 | The number of patients assigned to me exceeds my ability to provide adequate care | 1 | 0.000976563 | 1 | Excellent |
| 2 | I am faced with a lack of time to provide complete care services to patients. | 0.9 | 0.009765625 | 0.899013807 | Excellent |
| 3 | Due to lack of time, I am forced to prioritize patient care. | 0.9 | 0.009765625 | 0.899013807 | Excellent |
| 4 | In providing patient care, I am forced to ask for help from my colleagues. | 0.9 | 0.009765625 | 0.899013807 | Excellent |
| 5 | In providing patient care, due to lack of time, I entrust some care to the patient or their companion. | 1 | 0.000976563 | 1 | Excellent |
| 6 | I have enough time to provide complete education to the patient or their companion. | 1 | 0.000976563 | 1 | Excellent |
| 7 | Despite overlooking some care, I still do not have the ability to fully attend to all my patients. | 1 | 0.000976563 | 1 | Excellent |
| 8 | To gain time to perform medical care, I overlook some education for the patient and their companion. | 0.9 | 0.009765625 | 0.899013807 | Excellent |
| 9 | To attend to the patients under my care, I am forced to overlook some aspects of one patient's care to attend to another patient. | 1 | 0.000976563 | 1 | Excellent |
| 10 | After the end of my shift, I realize that I have forgotten to perform some care. | 0.8 | 0.043945313 | 0.790806946 | Excellent |
| 11 | I feel guilty for overlooking some services. | 1 | 0.000976563 | 1 | Excellent |
| 12 | To attend to all patients, I am forced to overlook some services. | 1 | 0.000976563 | 1 | Excellent |
| 13 | My nursing shifts each month are standard and proportionate to my ability level. | 0.9 | 0.009765625 | 0.899013807 | Excellent |
| 14 | Rationing nursing care contributes to an increase in my care-related errors. | 0.9 | 0.009765625 | 0.899013807 | Excellent |
| 15 | I cannot balance between patients who need more care and other patients. | 1 | 0.000976563 | 1 | Excellent |
| 16 | I am worried that I cannot fully perform all the care for my patients. | 1 | 0.000976563 | 1 | Excellent |
| 17 | I should allocate more time for each patient's nursing care, but the large number of patients does not allow it. | 0.9 | 0.009765625 | 0.899013807 | Excellent |
| 18 | I am forced to ration nursing care to distribute available resources fairly among patients. | 1 | 0.000976563 | 1 | Excellent |
| 19 | I have to make decisions based on the needs of the patients. | 0.9 | 0.009765625 | 0.899013807 | Excellent |
| 20 | Due to lack of skill in performing care correctly, I am forced to overlook some patient care. | 0.9 | 0.009765625 | 0.899013807 | Excellent |
| 21 | I am forced to ration nursing care based on time and resource limitations. | 0.9 | 0.009765625 | 0.899013807 | Excellent |
| 22 | While performing care, I feel that I have enough time to perform all the care for the patients. | 1 | 0.000976563 | 1 | Excellent |
| ***Note.*** ***I-CVI*** *= Item-Content Validity Index;* ***Pc*** *= Probability of chance agreement;* ***K***** = Modified Kappa Statistic. The interpretation in the 'Results' column is based on the K* value. K* values > 0.74 are considered excellent.* | | | | | |
