# Supplementary material for: Design and Psychometric Evaluation of a Nursing Care Rationing Instrument
Source: Nurs Open. 2026 Oct 16;13(10):e70933. doi: 10.1002/nop2.70933 (PMC13675515; doi:10.1002/nop2.70933)
Supplement: Supplementary file 5 [file NOP2-13-e70933-s005.doc]

| **Appendix Table S3**: Revised items in the qualitative content validity stage | | |
| --- | --- | --- |
| # | The initial item | The revised item |
| 1 | The number of patients under my care is more than my capacity. | The number of patients assigned to me exceeds my ability to provide adequate care |
